# Tree traits and canopy closure data from an experiment with 34 planted species native to Sabah, Borneo

**DOI:** 10.1016/j.dib.2015.12.048

**Published:** 2016-01-06

**Authors:** Malin Gustafsson, Lena Gustafsson, David Alloysius, Jan Falck, Sauwai Yap, Anders Karlsson, Ulrik Ilstedt

**Affiliations:** a Swedish University of Agricultural Sciences, Department of Forest Ecology and Management, 901 83 Umeå, Sweden; b Swedish University of Agricultural Sciences, Faculty of Natural Resources, Department of Ecology, 750 07 Uppsala, Sweden; c Conservation & Environmental Management Division, Yayasan Sabah Group, P.O. Box 11623, 88817 Kota Kinabalu, Sabah, Malaysia

## Abstract

The data presented in this paper is supporting the research article “Life history traits predict the response to increased light among 33 tropical rainforest tree species” [3]. We show basic growth and survival data collected over the 6 years duration of the experiment, as well as data from traits inventories covering 12 tree traits collected prior to and after a canopy reduction treatment in 2013. Further, we also include canopy closure and forest light environment data from measurements with hemispherical photographs before and after the treatment.

## 1 **Specifications table**

**Table:** 

Subject area	*Biology*
More specific subject area	*Tropical rainforest restoration and biodiversity*
Type of data	*Tables, figure*
How data was acquired	*Tree traits inventories; field surveys, wood density data from literature values, sulphuric acid–hydrogen peroxide procedure, atomic absorption spectrometer. Hemispherical photographs with the Canon 50D digital SLR camera and a Sigma EX DC 4.5 mm F2.8 circular 180° fisheye lens.*
Data format	*Raw*
Experimental factors	*34 native tree species (including 20 from the dipterocarp family) with 20 replicates each were planted in a lowland dipterocarp rainforest.*
Experimental features	*Tree height and mortality data was collected every year from planting in November 2008. A canopy reduction treatment was performed 50 months after planting. Tree species’ traits were noted at inventories 1 year before and after the treatment.*
Data source location	*Data collected in the Sg. Tiagau Forest Reserve (Tawau) in southeast Sabah, Malaysian Borneo (4°36’N, 117°12’E)*
Data accessibility	*Data is with this article*

## 2 **Value of the data**

• This data set on 12 traits for 34 dipterocarp and non-dipterocarp species native to Sabah, Borneo-can serve as benchmark data for conservation, ecological restoration and commercial forestry.
• Twenty randomized replications of each of the 34 species along an environmental gradient make the data interesting for studies on relationships between species traits and environmental factors, particularly to limiting light conditions at the rain forest floor.
• The study can also be used as model system for studies on ecological interactions and drivers for biodiversity. For example interactions between tree species and between tree species and other organisms, e.g. invertebrates or lichens.
• Future repeated yearly measurements will also make the study valuable for climate change effects on tree traits and phenology.

## 3 Data

This article is based on two kinds of data; tree species growth, survival and traits data, and canopy closure and forest light condition data before and after a canopy reduction treatment (Supplementary Table 1). The height growth and survival data was monitored every year over the 6 years duration of the experiment, while the species traits were measured on two occasions – one year before and after the canopy reduction treatment. The canopy closure and light environment in the forest were measured with the help of hemispherical photographs, which gave values of visible sky, leaf area index and global site factor. In-depth analyses of the data is presented in the associated research article [3].

## 4 Experimental design, materials and methods

### 4.1 Experimental design

The 3 ha experiment area is part of the INIKEA Sow-a-Seed Project situated in the Sg. Tiagau Forest Reserve in southeast Sabah (4°36’N, 117°12’E), Borneo (Supplementary Table 2). The site had been selectively logged during the 1970s when drought induced forest fires hit during the El Niño event in the early 1980s. The site had at the initiation of the experiment a secondary forest with a broken canopy which largely consisted of pioneer tree species (e.g. Macaranga), but also late successional species with some adult trees spread across the area.

The experiments’ planting material consisted of native seedlings germinated from seeds collected in the INIKEA Project area. The 34 presumed late successional species (Table 1) were selected from the INIKEA Project nursery based on the availability of enough stocking and of approximately even age at the time of the planting. The seedlings were transported to a second field nursery a month prior to planting for acclimatization. The species were randomly assigned to each planting point at the planting in November 2008, and dead seedlings were replaced at a second planting event one month later.

**Fig. 1:**
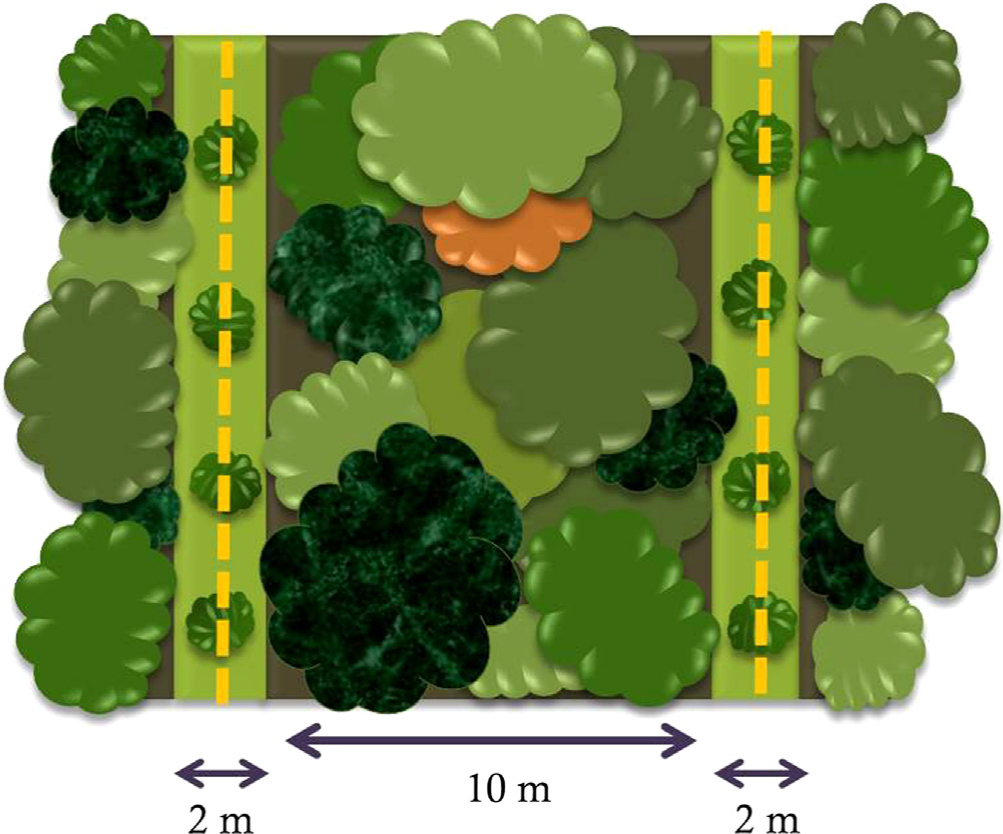
Line planting. A conceptual model of the line planting restoration method used in the Sow-a-Seed project in Sabah, Borneo. Lines were 2 m wide, with 10 m distance between the centers of adjacent lines. Competing vegetation was removed in the lines prior to planting, e.g. ground vegetation, bushes, small pioneer trees and climbers.

Trees were planted using a line planting method (Fig. 1) where 34 lines were created with conventional site preparation techniques, i.e. removing ground vegetation, climbers and pioneer saplings. The lines were 2 m wide with 8 m secondary forest between lines. Along each line 20 planting points were established, and points that were judged to be unpalatable (e.g. because of rocks or a landslide) were avoided in favor for the succeeding point. Maintenance (i.e. weeding, girdling and cutting climbers and pioneers species saplings) was conducted when necessary – about 1–3 times per year – to keep the lines free from competing vegetation. About four years after planting we conducted a canopy reduction treatment to increase the forest light conditions up to levels present in tree gaps. It was performed similar to conventional tropical forest restoration operations, where it is common to conduct a treatment a few years after planting where surrounding vegetation is reduced to give more light to the planted trees.

### 4.2 Tree species data

Height and survival data was collected 6 months after planting, and then from 12 months after planting on a yearly basis. Height was measured from the stem base to the tree top. The tree species traits inventories were conducted 1 year before and after the canopy reduction treatment, and 12 traits were included; height growth (HG) response, upper quartile HG response, early HG, mortality rate, wood density, specific leaf area, crown depth, crown width, leaved stem length, and nitrogen (N), phosphorous (P) and potassium (K) content in leaves.

The response data was calculated as: HG during the year following the canopy reduction treatment reduced from HG during the year prior to treatment. Early HG was calculated as: HG at 12 months reduced from HG at 6 months. Mortality rate (*M*) was calculated as: (log[*N* at planting]−log[*N* at 40 months])/time [5]. The wood density data was literature values from the World Agroforestry Centre Wood Density Database [6] (Supplementary Table 3). Specific leaf area was calculated as the leaf area per unit leaf dry weight; where the dry weight came from oven drying and weighing the leaves, and the area was estimated from leaf scans with the Digimizer software (http://www.digimizer.com). The crown depth was the stem height reduced from the tree height, and the crown width was measured from the widest part of the crown in two orthogonal directions. Leaved stem length was measured as the extension growth from the lowest leaf growing directly from the branch to the axis tip [4].

The leaf content nutritional values were sampled from the post canopy reduction treatment inventory and sent to the Sabah Forestry Department laboratory in Sepilok, Sandakan for determination of total N, P, and K content. The samples were digested following the sulfuric acid–hydrogen peroxide procedure [1], and the results are based on air-dry weight (50 °C to constant weight). N content was measured with the auto-analyzer (Burkard SFA2, UK). P content was determined using the molybdenum-blue method [2] with readings from a wavelength of 880 nm on the spectrophotometer (HITACHI UV–vis, Japan). K content was measured with the atomic absorption spectrometer (GBC, Australia).

### 4.3 Canopy reduction treatment

We performed a canopy reduction treatment with the aim to increase the light that reaches our planted seedlings. Competing vegetation was removed, climbers and lianas were cut and saplings and small trees of pioneer species were girdled. The light environment at each planting point was measured with hemispherical photographs at two occasions – one year before and after treatment – using a Canon 50D digital SLR camera with a Sigma EX DC 4.5 mm F2.8 circular 180° fisheye lens. Photos of the canopy were taken with the camera facing upwards from 1.3 m above the ground, with the help of a monopod with a self-levelling and stabilizing mount (SLM8 from Delta-T), at 1.5 m distance from a planting point in the two directions along the line. We collected data during the early morning, from 30 min after sunrise to 9 am to obtain good quality photos in which sky and canopy were clearly distinguishable. Average values for the two photos were used in the data analysis. The Delta-T HemiView software was used to analyze the hemispheric photos to obtain data on the proportion of visible sky, leaf area index (LAI), and global site factor (GSF) (Supplementary Table 4).

## Figures and Tables

**Table 1:** Scientific names and species codes for 34 native tree species planted in Sabah, Borneo.

Scientific name	Code

**Dipterocarpaceae**	
*Dipterocarpus conformis*	dico
*Dryobalanops keithii*	drke
*Dryobalanops lanceolata*	drla
*Hopea ferruginea*	hofe
*Parashorea malaanonan*	pama
*Parashorea smythiesii*	pasm
*Parashorea tomentella*	pato
*Shorea beccariana*	shbe
*Shorea falciferoides*	shfa
*Shorea fallax*	shfx
*Shorea leprosula*	shle
*Shorea leptoderma*	shlt
*Shorea macrophylla*	shma
*Shorea macroptera*	shme
*Shorea ovalis*	shov
*Shorea parvifolia*	shpa
*Shorea pauciflora*	shpu
*Shorea platyclados*	shpl
*Shorea sp.*	shsp
*Shorea xanthophylla*	shxa
	
**Other climax species**	
*Baccaurea angulata*	baan
*Baccaurea* sp.	basp
*Canarium* sp.	casp
*Diospyros* sp.	disp
*Durio* spp.	dusp
*Heritiera* sp.	hesp
*Intsia palembanica*	inpa
*Koompasia excelsa*	koex
*Mangifera odorata*	maod
*Mangifera panjang*	mapa
*Pentace adenophora*	pead
*Pentace laxiflora*	pela
*Sindora iripicina*	siir
*Walsura pinnata*	wapi

## Footnotes

Appendix A Supplementary data associated with this article can be found in the online version at doi:10.1016/j.dib.2015.12.048.
