# Supplementary material for: Tree traits and canopy closure data from an experiment with 34 planted species native to Sabah, Borneo
Source: Data Brief. 2016 Jan 6;6:466–70. doi: 10.1016/j.dib.2015.12.048 (PMC4716456; doi:10.1016/j.dib.2015.12.048)
Supplement: Supplementary file 1 — Supplementary material [file mmc1.docx]

Dear Editor,

There are no Conflicts of Interests for the manuscript DIB-D-15-00596.

Thank you.

Kind regards,

Malin Gustafsson
